# Survival and prognostic factors in mucosal melanomas of the oral cavity: A meta-analysis

**DOI:** 10.34172/joddd.025.41492

**Published:** 2025-06-30

**Authors:** Alberto Rodriguez-Archilla, Pablo Valverde-Martinez

**Affiliations:** ^1^Department of Stomatology, Oral Medicine Unit, Faculty of Dentistry, University of Granada, Granada, Spain; ^2^Biohealth Research Institute (IBS), Granada, Spain

**Keywords:** Melanoma, Mouth, Prognosis, Risk factors, Survival

## Abstract

**Background.:**

The development of early metastases due to the high vascularization and lymphatic drainage of the oral cavity, followed by delayed diagnosis, results in a worse prognosis of oral melanomas. The present study aimed to determine the survival of oral melanoma in different periods and analyze its prognostic factors.

**Methods.:**

A search for studies on survival and prognostic factors of oral melanoma was performed in the following databases: PubMed (MEDLINE, Cochrane Library), Web of Science (WoS), and Scopus. The estimation of the pooled proportion was carried out with the generic inverse variance method, using the standard error (SE) of the proportion. For dichotomous outcomes, the estimates of the effects of the intervention were expressed as odds ratios (ORs) using the Mantel-Haenszel (M-H) method, all with 95% confidence intervals.

**Results.:**

Thirty-eight studies that considered 3767 oral melanoma patients were included in this meta-analysis. Overall survival (OS) decreased from 58% at two years to 42% at three years and 29% at five years. Regarding prognostic factors, non-ulcerated oral melanomas with a high degree of pigmentation showed the best survival at 5 years. In contrast, oral tumor location and gender did not significantly affect oral melanoma survival.

**Conclusion.:**

Oral melanoma has a low survival rate, with ulcerated and poorly pigmented tumors having the worst prognosis.

## Introduction

 Oral melanomas account for approximately 4% of all melanomas. They are mainly diagnosed between the 4th and 7th decades of life, with a peak incidence around 60 years of age, and are slightly more prevalent in males. Unlike cutaneous melanomas, the incidence of oral melanoma has remained stable in recent years.^1^ Its etiology remains unknown, although some risk factors, such as chronic inflammation induced by smoking or chronic mechanical irritation, have been suggested. Notably, a significant proportion of oral melanomas arise de novo from the apparently normal oral mucosa. However, approximately 30%‒40% of cases are preceded by oral pigmentations that persist for several months or even years.^2^ The main oral locations for melanomas are the palate and the alveolar gingival ridge. The lack of symptomatology associated with oral melanoma often results in patients delaying medical attention, which can lead to delayed diagnosis and a poor prognosis. Early and prompt diagnosis of oral melanomas is vital for the patient’s prognosis, as they exhibit more aggressive behavior and worse prognosis than melanomas with other locations in the body.^3^ Indeed, almost one-third of patients present with lymph node metastases at the time of diagnosis of primary oral melanoma. The early development of metastases may be attributed to the high level of vascularization and lymphatic drainage in the oral cavity.^4^ This study aimed to determine the survival of patients with oral melanoma in different periods and assess its prognostic factors.

## Methods

 All research steps (search, study selection, and data extraction) were achieved independently by both authors (ARA and PVM). Discrepancies in article selection were resolved by consensus.

 The research question was: How do different prognostic factors influence the survival of patients with oral melanoma?

###  Search strategy

Table 1 shows the search strategies in each database using a combination of Medical Subject Headings (MeSH) and free-text terms. The inclusion criteria were as follows: a) all types of articles related to our purpose and b) articles written in any language and with no restrictions on publication date. The exclusion criteria were: a) articles with no full-text availability, b) articles with a relevant risk of bias (score ≤5 stars on the Newcastle-Ottawa methodological quality assessment scale),^5^ c) articles with no clinical data, and d) studies with non-usable data.

**Table 1:** Search strategies for the three databases

**Database**	**#**	**Search strategy**	**Results**
PubMed	#1	“melanoma”[MeSH Terms]	112,400
	#2	“mouth”[MeSH Terms]	326,150
	#3	#1 AND #2	619
	#4	(“survival”[MeSH Terms] OR “prognosis”[MeSH Terms])	1,990,947
	#5	#3 AND #4	134
Web of Science (WoS)	#6	(“melanoma”[Topic] AND “mouth” [Topic])	834
	#7	(“survival” [Topic] OR “prognosis” [Topic])	1,244,600
	#8	#6 AND #7	247
Scopus	#7	TITLE-ABS-KEY (“melanoma” AND “mouth”)	2,718
	#8	TITLE-ABS-KEY (“survival” OR “prognosis”)	2,322,214
	#9	#7 AND #8	227

###  Assessment of methodological quality

 The methodological quality of the articles was screened using the Newcastle-Ottawa (NOS) methodological quality assessment scale,^5^ which is composed of eight items that evaluate three dimensions (selection, comparability, and exposure). Considering the score obtained, the studies are classified as high quality (≥7 stars), moderate quality (4‒6 stars), and low quality (0‒3 stars).

###  Data extraction

 Overall survival (OS) of oral mucosal melanomas was established in three periods: 2, 3, and 5 years. Prognostic factors related to survival, such as the oral tumor location, the gender of the patients, the existence of ulceration of the lesion, or the degree of pigmentation of the neoplasm, were also evaluated.

###  Statistical analysis

 For the meta-analysis, data were processed with RevMan 5.4 software (The Cochrane Collaboration, Copenhagen, Denmark). The proportion (P) was calculated by dividing the number of positive cases (n) by the total population (N). Estimation of the proportion was carried out with the generic inverse of variance method, using the standard error (SE) of the proportion and 95% confidence intervals (95% CI). The SE was obtained using the formula SQRT (P*(1-P)/N). For dichotomous outcomes, the odds ratio (OR) with the Mantel-Haenszel chi-squared formula (M-H) was used, both with 95% confidence intervals (95% CI). Heterogeneity was determined according to the Higgins statistic (I^2^). The random-effects model was applied in cases of high heterogeneity (I^2^>50%). A P-value below 0.05 was considered the minimum level of significance. The risk of publication bias was assessed using MedCalc Statistical Software version 23.1.7 (MedCalc Software Ltd., Ostend, Belgium). A funnel plot and Egger’s regression test were employed, with a minimum of 10 studies required for analysis. Publication bias was considered present if asymmetry was observed in the funnel plot and if Egger’s test yielded a *P* value<0.05.

## Results

###  Study selection

**Figure 1:**
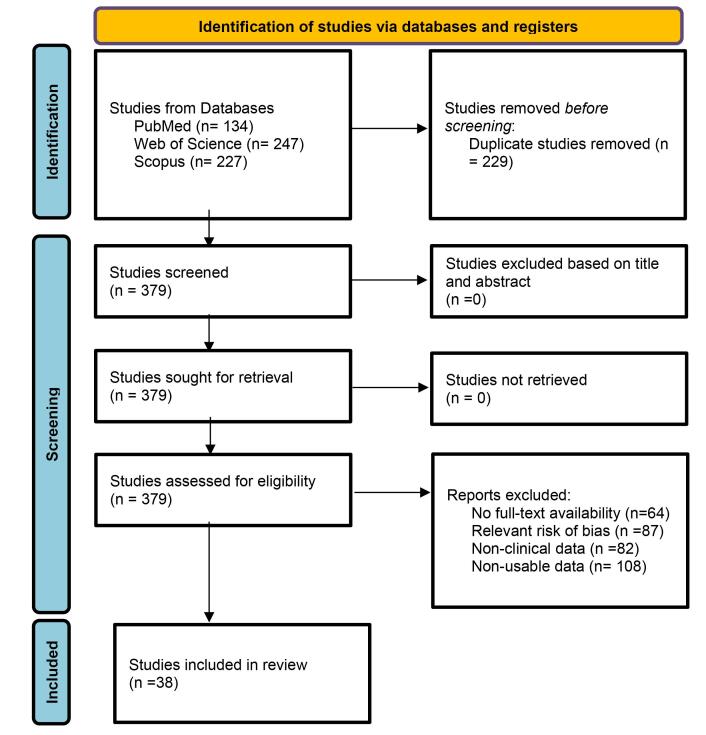
Study selection flowchart

 In the initial search, 608 articles were found (134 in PubMed, 247 in WoS, and 227 in Scopus) between 1955 and 2021, with 229 duplicates, leaving 379 eligible articles. A total of 341 studies were excluded due to a) articles with no full-text availability (n=64), b) articles with a relevant risk of bias (score ≤5 stars on the NOS) (n=87), c) articles without clinical data (n=82), and d) studies with non-usable data (n=108). After applying these criteria, 38 studies were included in this meta-analysis (Figure 1).

Table 2 summarizes the main descriptive characteristics and the methodological quality of 38 studies^6-43^ included in this meta-analysis, as assessed using the Newcastle-Ottawa Scale (NOS). A total of 3767 patients with oral mucosal melanoma were analyzed, with 50.4% males and 49.6% females. The studies were conducted in the following countries: United States of America (12 studies), China (8), Brazil (3), Japan (3), Denmark (2), France (2), Germany (2), Australia (1), Canada (1), Italy (1), South Korea (1), The Netherlands (1), and Turkey (1). According to the NOS, nine studies (23.7%) received 6 stars (moderate quality), 21 studies (55.3%) received 7 stars (high quality), and 8 studies (21.0%) received 8 stars (high quality).

**Table 2:** Descriptive characteristics and methodological quality evaluation of the 38 studies included in this meta-analysis

**Study, year**	**Country**	**Study populations (gender distribution, mean age)**	**Other parameters assessed**	**NOS**
Abraham, 2010^6^	USA	27 pat. (15M, 12F; 68y)	Race, treatment, stage, follow-up, OS	6
Bakkal, 2015^7^	Turkey	10 pat. (6M, 4F; 66y)	Treatment, stage, follow-up, local recurrence, regional recurrence, OS	6
Berthelsen, 1984^8^	Denmark	38 pat. (31M, 7F; 64y)	Treatment, stage, follow-up, OS	7
Breik, 2016^9^	Australia	14 pat. (8M, 6F; 65y)	Treatment, pathology, stage, follow-up, OS	6
Chae, 2020^10^	South Korea	74 pat. (41M, 33F; 59y)	Treatment, bone invasion, resection margin status, stage, follow-up, OS	8
Conley, 1974^11^	USA	52 pat. (36M, 16F; 60y)	Treatment, stage, follow-up, OS	8
Francisco, 2016^12^	Brazil	51 pat. (31M, 20F; 59y)	Race, treatment, stage, recurrence, follow-up, OS	8
Guo, 2020^13^	China	92 pat. (40M, 52F; na)	Treatment, pigmentation, stage, recurrence, follow-up, OS	6
Jethanamest, 2011^14^	USA	815 pat. (382M, 433F; na)	Race, treatment, stage, follow-up, OS	7
Lawaetz, 2016^15^	Denmark	98 pat. (41M, 57F; na)	Treatment, stage, pigmentation, ulceration, follow-up, OS	7
Lian, 2017^16^	China	706 pat. (233M, 473F; 55y)	Treatment, stage, BRAF mutation, cKIT mutation, follow-up, OS	7
Meleti, 2008^17^	The Netherlands	14 pat. (5M, 9F; 57.9y)	Treatment, stage, follow-up, OS	6
Moore, 1955^18^	USA	26 pat. (na, na; na)	Race, treatment, stage, follow-up, OS	6
Moya-Plana, 2019^19^	France	45 pat. (31M, 14F; 64y)	Treatment, stage, margins, follow-up, OS	7
Naganawa, 2016^20^	Japan	19 pat. (9M, 10F; na)	Treatment, stage, follow-up, OS	6
Owens, 2003^21^	USA	48 pat. (39M, 9F; 55.5y)	Treatment, stage, follow-up, OS	7
Patel, 2002^22^	USA	59 pat. (34M, 25F; 63y)	Treatment, stage, vascular invasion, follow-up, OS	8
Perri, 2017^23^	Italy	20 pat. (14M, 6F; 54y)	Treatment, stage, pigmentation, ulceration, follow-up, OS	6
Prasad, 2002^24^	USA	40 pat. (30M, 10F; 59.5y)	Treatment, stage, pigmentation, ulceration, follow-up, OS	8
Prinzen, 2019^25^	Germany	50 pat. (25M, 25F; 65y)	Treatment, stage, sentinel node biopsy, follow-up, OS	7
Rapini, 1985^26^	USA	124 pat. (78M, 46F; na)	Treatment, stage, follow-up, OS	7
Sahovaler,2021^27^	Canada	76 pat. (33M, 43F; 66.3y)	Treatment, stage, margins, follow-up, OS	7
Schaefer, 2017^28^	Germany	32 pat. (22M, 10F; 64.5y)	Treatment, stage, follow-up, OS	7
Schmidt, 2017^29^	USA	326 pat. (180M, 146F; 66y)	Treatment, stage, neck dissection, follow-up, OS	7
Shah, 1977^30^	USA	74 pat. (47M, 27F; na)	Race, treatment, stage, follow-up, OS	7
Shuman, 2011^31^	USA	52 pat. (21M, 31F; 66y)	Treatment, stage, margins, follow-up, OS	7
Soares, 2021^32^	Brazil	8 pat. (6M, 2F; 53.6y)	Treatment, stage, pigmentation, biomarkers, follow-up, OS	6
Song, 2016^33^	China	62 pat. (34M, 28F; 55.4y)	Treatment, stage, follow-up, OS	8
Song, 2017^34^	China	62 pat. (34M, 28F; 55.4y)	Treatment, stage, BAP1 expression, follow-up, OS	7
Sun, 2012^35^	China	51 pat. (36M, 15F; na)	Treatment, stage, pigmentation, neck dissection, follow-up, OS	7
Tanaka, 2004^36^	Japan	35 pat. (14M, 21F; 65.2y)	Treatment, stage, follow-up, OS	8
Temam, 2005^37^	France	69 pat. (36M, 33F; na)	Treatment, stage, follow-up, OS	7
Trodahl, 1970^38^	USA	42 pat. (36M, 6F; 49.5y)	Treatment, stage, follow-up, OS	7
Wang, 2013^39^	China	81 pat. (50M, 31F; na)	Treatment, stage, heparanase expression, follow-up, OS	7
Wu, 2018^40^	China	170 pat. (91M, 79F; na)	Treatment, stage, ulceration, follow-up, OS	8
Yamada, 2017^41^	Japan	38 pat. (24M, 14F; 65.2y)	Treatment, stage, follow-up, OS	7
Yang, 2010^42^	China	78 pat. (50M, 28F; 53.8y)	Treatment, stage, recurrence, follow-up, OS	7
Yii, 2003^43^	UK	89 pat. (43M, 46F; 64y)	Race, treatment, stage, follow-up, OS	7

NOS: Newcastle-Ottawa methodological quality scale; pat.: patients with oral mucosal melanoma; M: male; F: female; y: mean age in years; na: data not available; OS: overall survival.

###  Overall survival of oral melanoma at 2 years

**Figure 2:**
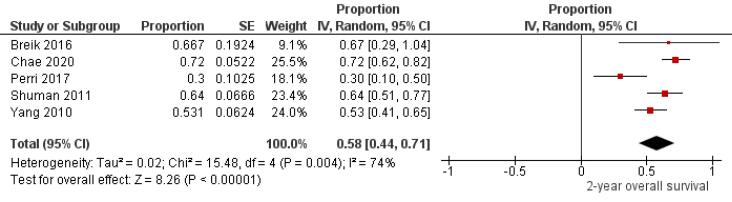
Studydata and forest plot graph for 2-year overall survival of patients with oral melanoma. SE: standard error

 Five studies^9,10,23,31,42^ that included 216 patients with oral melanoma (Figure 2) found a pooled 2-year OS of 58% (95% CI: 44% to 71%) with high heterogeneity between studies (I^2^: 74%). Study variability ranged from a maximum OS of 72% (95% CI: 62% to 82%)^10^ to a minimum OS of 30% (95% CI: 10% to 50%).^23^

###  Overall survival of oral melanoma at 3 years

**Figure 3:**
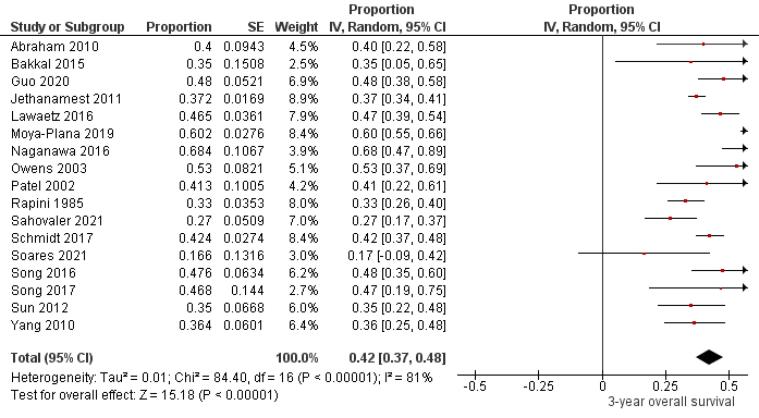
Study data and forest plot graph for 3-year overall survival of patients with oral melanoma. SE: standard error

 Seventeen studies,^6,7,13-15,19-22,26,27,29,32-35,42^ including 837 oral melanoma patients (Figure 3), found a pooled 3-year OS of 42% (95% CI: 37% to 48%) with high heterogeneity between studies (I^2^: 81%). Study variability ranged from a maximum OS of 68% (95% CI: 47% to 89%)^20^ to a minimum OS of 17% (95% CI: 0% to 42%).^32^

###  Overall survival of oral melanoma at 5 years

**Figure 4:**
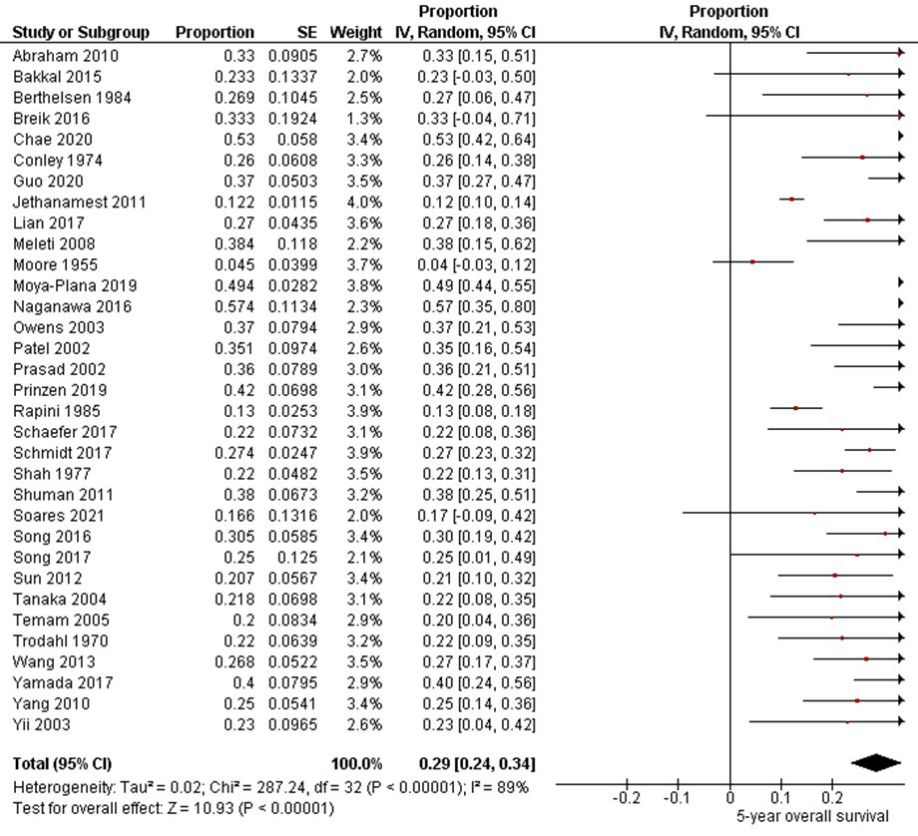
Study data and forest plot graphs for 5-year overall survival of patients with oral melanoma. SE: standard error

 Thirty-three studies^6-11,13,14,16-22,24-26,28-39,41-43^ involving 2773 patients with oral melanoma (Figure 4) found a pooled 5-year OS of 29% (95% CI: 24% to 34%) with high heterogeneity between studies (I^2^: 89%). Study variability ranged from a maximum OS of 57% (95% CI: 35% to 80%)^20^ to a minimum OS of 4% (95% CI: 0% to 12%).^18^

###  Overall survival of oral melanoma related to other clinical parameters

Table 3 shows the possible influence of other clinical parameters (oral location of the tumor, gender, ulceration, and the degree of pigmentation) on the OS of oral mucosal melanomas.

**Table 3:** Analysis of different clinical parameters related to 5-year survival of oral melanomas

**Parameter**	**References**	**Value**	**OR**	**[95% CI]**	**I**^2^** (%)**	***P***
Oral location of melanoma	^17,39,42^	Palate	1.49	[0.58 to 3.81]	0%	0.41
Gender	^17,26,33,39,42^	Female	1.41	[0.91 to 2.18]	0%	0.13
Ulceration	^24,25,42,43^	No	3.78	[2.24 to 6.39]	0%	<0.001*
Degree of pigmentation	^13,24-26,39^	Highly pigmented	2.47	[1.46 to 4.19]	0%	<0.001*

OR: odds ratio; CI: confidence interval; I^2^ (%): Higgins statistic for heterogeneity (percentage); *Statistically significant.

 Three studies^20,39,40^ evaluated the oral location of the melanoma, observing a higher probability of 5-year survival in tumors located in the palate, although without reaching statistical significance (OR=1.49, 95% CI: 0.58 to 3.81, *P*=0.41). Five studies^20,25,35,39,40^ focused on the patient’s gender. Although females exhibited a longer 5-year survival compared to males, the results were also not statistically significant (OR=1.41, 95% CI: 0.91 to 2.18, *P*=0.13).

 Four studies^12,33,34,40^ compared oral ulcerated and non-ulcerated melanomas concerning 5-year survival. Patients with non-ulcerated melanomas were 3.78 times more likely to be alive at 5 years with a highly significant association (OR=3.78, 95% CI: 2.24 to 6.39, *P*<0.001).

 Five studies^13,33-35,39^ examined the degree of pigmentation of melanomas. Patients with highly pigmented tumors were 2.47 times more likely to be alive at 5-year follow-up. After statistical analysis, highly significant differences were found (OR=2.47, 95% CI: 1.46 to 4.19, *P*<0.001).

###  Publication bias

Table 4 presents the assessment of publication bias based on Egger’s regression test. Publication bias was confirmed by the presence of asymmetry in the funnel plot with *P*<0.05 in Egger’s test for 5-year OS (*P*=0.0029) but not for 3-year OS (*P*=0.8653).

**Table 4:** Analysis of publication bias according to Egger’s regression test

**Parameter**	n*	**t**	**[95% CI]**	***P*** ** value**
OS oral melanoma at 2 years	5	NA	NA	NA
OS oral melanoma at 3 years	17	0.1760	[-1.9971 to 2.3490]	0.8653
OS oral melanoma at 5 years	33	2.3638	[0.8710 to 3.8566]	0.0029
Oral location of melanoma	3	NA	NA	NA
Gender	5	NA	NA	NA
Ulceration	4	NA	NA	NA
Degree of pigmentation	5	NA	NA	NA

* Minimum of 10 studies to perform the analysis; OS: overall survival; n: number of studies; t: intercept; CI: confidence interval; NA: not assessable.

## Discussion

 The present meta-analysis on survival and prognostic factors related to oral melanoma included data from 38 studies.

 In this study, the 2-year survival of oral melanomas was 58%. Of the five studies that analyzed this parameter, four^9,10,31,42^ reported percentages similar to the current paper, whereas one study^23^ found a considerably lower survival (30%). In the present study, the 3-year survival of oral melanomas reached 42%. Of the seventeen studies that evaluated this variable, thirteen^6,7,13-15,21,22,26,29,33-35,42^ found similar percentages, two^19,20^ found significantly higher percentages (>60%), and two other studies^27,32^ found much lower survival percentages (<30%). In this study, the 5-year survival rate for oral melanoma was 29%. Of the 35 studies examining this parameter, 18 studies^6-9,11,16,22,24,28-30,32,33,36,38,39,42,43^ reported a 5-year survival percentage similar to that of this study (range: 22‒36%); seven studies^13,17,19-21,25,41^ obtained a relatively higher survival (>37%), and six others^14,18,26,32,35,37^ reported significantly lower 5-year survival percentages (<21%).

 Oral melanomas carry a worse prognosis than cutaneous melanomas due to their propensity for local invasion and distant metastasis. This is primarily attributed to delayed diagnosis, atypical clinical presentation, and continuous trauma related to their anatomical location, often resulting in ulcerated lesions as the disease progresses.^44^ Resection of primary oral melanoma lesions is the gold standard of treatment for these lesions. The main problem stems from the early metastatic nature of oral melanomas. Lymph node metastases are common, and neck dissection does not affect the development of future distant metastases or OS. Survival of patients with oral melanoma varies according to the tumor site and decreases when brain or liver metastases are present. The primary site of oral melanoma location, bone invasion, resection margins, depth of tumor invasion, and distant metastases are critical factors in predicting prognosis. They should be considered when selecting the most appropriate therapeutic option for treating oral mucosal melanoma.^10^ Other factors with a relevant influence on oral melanoma survival are the type of melanoma, with amelanotic melanomas having the worst prognosis probably due to diagnostic delay^32^ or the overexpression of certain genes such as the BAP1 gene that regulates cell differentiation, division, and death.^34^ The BAP1 gene encodes a tumor suppressor protein that plays a pivotal role in the pathogenesis of uveal melanoma. However, its relevance in oral melanomas remains poorly understood. In uveal melanoma, high BAP1 expression has been significantly associated with a favorable clinical prognosis. Conversely, in oral melanomas, BAP1 overexpression appears to be linked to an adverse prognosis and decreased OS. Evidence suggests that BAP1 inactivation in oral mucosal melanomas may play a role in tumor progression by promoting a more aggressive biological phenotype and reduced patient survival.^45^ Similarly, overexpression of heparanase, an enzyme released in the metabolism of tumor cells, also leads to poorer survival in oral melanomas, as they have a worse biological behavior.^39^ In oral mucosal melanomas, the transition from the radial growth phase to the invasive phase represents a critical step in tumor progression, as malignant cells migrate and infiltrate the underlying connective tissue. This process involves the synthesis and release of enzymes required for extracellular matrix degradation, such as heparanase.^46^

 In the present study, melanomas located on the palate had a higher probability of 5-year survival, although statistical significance was not reached (*P*=0.41). Two^39,40^ of the three studies that considered tumor location coincided in indicating the palatal location, while the other one^20^ did not. This longer survival in tumors located in the palate could be conditioned by the achievement of negative resection margins at the time of surgery. This is much more difficult in other melanoma sites, such as the pharynx or paranasal sinus.^20^

 Although women had a longer 5-year survival than men in this work, the results were also not statistically significant (*P*=0.13). Of the five studies that focused on the patient’s gender, four^12,20,25,35^ confirmed this longer survival in females, while for one,^39^ it was longer in males. The reason for the shorter survival of males with oral melanoma may lie in the higher incidence of this malignant tumor in males. The actual influence of gender on the biological behavior of oral mucosal melanomas remains to be elucidated.^39^ Women’s greater awareness of health problems may lead to an earlier diagnosis of oral melanoma and a longer survival time.^25^

 In the present work, non-ulcerated melanomas increased the probability of 5-year survival by 3.78 times, with highly significant statistical differences (*P*<0.001). All studies^12,33,34,40^ that further examined this parameter corroborated this increased survival in non-ulcerated lesions. Ulceration is a clinical sign of long-term lesions found in the more advanced stages (III or IV) of the disease. These melanomas have a higher degree of invasion and deep infiltration that drastically decreases survival.^40^

 In this study, patients with more pigmented melanomas were 2.47 times more likely to be alive at 5-year follow-up, with a highly significant statistical association (*P*<0.001). All studies^13,33-35,39^ that looked at the degree of pigmentation agreed that the more pigmented the melanoma, the higher the probability of 5-year survival. Due to their clinical presentation as red-like lesions, poorly pigmented melanomas (amelanotic melanomas) present delays in diagnosis. There is often diagnostic confusion with lesions of inflammatory-infectious origin. The diagnosis is made later, as they are metastatic lesions with a worse prognosis.^13^

## Limitations

 This study had some limitations. It was not possible to properly analyze certain parameters that significantly influence survival in oral melanomas, such as the evolution time of the tumor lesion or the presence of lymph nodes and distant metastases. Nor was it possible to evaluate the impact on survival of the different treatment alternatives of oral melanomas. Finally, the significant variability between studies may have conditioned the results, requiring a cautious interpretation.

## Conclusion

 In this meta-analysis, the OS of oral mucosal melanoma patients decreased from 58% at two years to 42% at three years and 29% at five years. Considering the prognostic factors, non-ulcerated oral melanomas with a high degree of pigmentation showed the highest 5-year survival. In contrast, oral tumor location and gender did not significantly affect oral melanoma survival.

## Competing Interests

 None.

## Ethical Approval

 Granada University Stomatology Department Scientific Committee (code EST/UGR/19-2024).
